# Chemical and biological language models in molecular design: opportunities, risks and scientific reasoning

**DOI:** 10.2144/fsoa-2023-0318

**Published:** 2024-05-15

**Authors:** Jürgen Bajorath

**Affiliations:** 1 Department of Life Science Informatics & Data Science, B-IT, LIMES Program Unit Chemical Biology & Medicinal Chemistry, Lamarr Institute for Machine Learning & Artificial Intelligence, Rheinische Friedrich-Wilhelms-Universität, Friedrich-Hirzebruch-Allee 5/6, D-53115 Bonn, Germany

**Keywords:** artificial intelligence, chemical language models, drug discovery, model explanation, protein language models, sequence-to-compound learning

In the physical and life sciences, including drug discovery, the use of deep learning (DL) models, such as language models (LMs) or graph neural networks (GNNs), is on the rise for various applications. LMs are generally designed to translate sequences of characters and are particularly versatile and adaptable to many different machine translation tasks, giving rise to their popularity in many areas. Transformer networks, with their multi-head self-attention mechanism, and encoder–decoder frameworks [1] have become powerful LMs for many applications. However, the versatility of DL architectures comes at a price. While they open the door to novel applications, their use is also prone to misconceptions or misunderstandings, often leading to false assumptions and controversial views of scientific applications. This commentary discusses the general requirements, potential caveats or pitfalls and explanation of DL models in the context of molecular design.

## Model explanation & implications

In the realm of complex DL models, characterized by their ‘black box’ nature, procedures providing insights into their operations and predictions are crucial for avoiding incorrect expectations or questionable conclusions. In machine learning (ML) including DL, it is encouraging to note increasing application of approaches for model explanation including feature attribution methods such as Shapley additive explanations (SHAP) [2,3]. Such methods belong to explainable artificial intelligence (XAI) and are used to analyze model decisions and explain predictions. Model-agnostic methods such as SHAP are particularly useful since they can be applied to different ML models and enable comparison of their prediction characteristics. There are also other approaches for shedding light on black boxes and helping to rationalize predictions. For example, for transformers, attention weights can be visualized in feature maps to identify features driving predictions. However, a misunderstanding in model explanation is considering the results of feature attribution analysis as a chemical or biological interpretation. Any feature attribution methodology aims to identify features that determine a prediction. Importantly, identifying such features does not ensure interpretability. The question if identified key features might be chemically or biologically intuitive and interpretable must be subsequently addressed. For example, structural features driving correct predictions of active compounds can be mapped on test compounds and visualized. This additional analysis step makes it possible to determine if key features form coherent substructures that might be associated with the biological activity of test compounds [3,4]. Features determining predictions might not always be understandable based on human reasoning because the decisions of ML models are statistically determined. Thus, the potential lack of feature interpretability is not a shortcoming of correctly used feature attribution or visualization methods. This is not always considered when attempting to rationalize predictions, reflecting a misconception.

In a similar vein, the fundamental distinction between correlation and causality in ML [5] is often not taken into consideration. For example, in activity prediction, structural features shared by training and test compounds might strongly correlate with prediction accuracy. However, correlation in ML does not ensure causality. In this example, causality would apply if characteristic structural features would not only determine prediction accuracy but also be directly responsible for the given biological activity. However, this is a different question. For instance, the presence of structural features distinguishing active from inactive compounds might be coincidental or result from data bias. Accordingly, one might hypothesize causality in light of accurate predictions, but firmly establishing causality would require experimental work further investigating these features. For many ML applications in the life sciences, testing causality hypotheses requires experimental follow-up.

## Model-dependent risks

A major attraction of GNNs, transformers, or other DL models is their potential to address prediction tasks that were previously unfeasible. However, when addressing novel prediction tasks, there is the potential of confusion because accurate predictions might be obtained for other than apparent or expected reasons, thus representing ‘Clever Hans' incidences [6,7]. This name originates from the true story of a horse (named Hans) that was long believed to be able to count, until artifacts were uncovered [7]. In ML, Clever Hans effects have often been identified retrospectively. For example, in drug discovery, accurate predictions of the binding affinity or relative free energies of active compounds continue to be challenging [8]. GNNs have recently been used for compound affinity predictions based on graph representations of protein–ligand interactions extracted from x-ray structures. These studies typically produced fairly accurate predictions, leading to conclusions that GNNs are capable of learning protein–ligand interactions and quantifying binding energies. However, detailed control calculations and XAI analysis demonstrated subsequently that these predictions were largely determined by ligand memorization effects [9,10]. Similar compounds often bind to the same or related targets with comparable potency. Therefore, depending on the composition of training and test sets, reasonable predictions might be obtained if GNNs memorized similar compounds and their affinity. Hence, the results of affinity predictions using GNNs did not depend on learning protein–ligand interactions, representing an exemplary Clever Hans effect. It follows that special care must be taken in exploring novel prediction scenarios with black box DL models. Formulating a clear hypothesis that can be directly tested by a ML model and appropriate controls often helps to avoid Clever Hans effects and incorrect conclusions [11].

## Focus on language models

Models originating from natural language processing are increasingly employed for machine translation tasks in other fields. LMs consist of recurrent neural networks (RNNs) or transformers, which are increasingly used, and are particularly versatile in learning translations of different types of sequential or textual data representations. Small molecules are typically encoded as string representations. For instance, in the life sciences and drug discovery, LMs can be trained to learn compound-to-compound, protein-to-protein, or protein-to-compound mappings. In generative compound design, this makes it possible to predict new compounds from reference molecules or protein sequence data. LMs for compound-to-compound learning are often referred to as ‘Chemical LMs' (CLMs) while LMs for supervised or unsupervised learning from protein sequences are referred to as ‘Protein LMs' (PLMs).

## Sequence-based compound design

Compound design based on reference molecules is a standard approach in drug design that is not limited to LMs, but feasible with a variety of computational methods. By contrast, the prediction of new active compounds from protein sequences is difficult, if not impossible using other computational approaches. Accordingly, attempts have recently been made to distinguish true protein–ligand pairings (complexes) from randomly assembled (false) pairings [^12-15^] or to predict compounds from protein sequence data directly [^15-18^]. For these and other applications, PLMs are also used for representation learning from amino acid sequences, yielding sequence embeddings that implicitly capture structural and functional characteristics of proteins [19,20]. For predicting protein–ligand pairings using LMs, tokenized amino acid sequence and compound representations are combined. Potential applications of these models include target validation (for example, for active compounds from phenotypical screens) or compound repurposing (finding alternative targets and applications for active compounds or drugs). For the prediction of new compounds from protein sequences, representations such as protein embeddings serve as input for generating compound (output) strings. Models for the assessment of protein-ligand pairs or sequence-based compound predictions typically combine PLM and CLM components. Such LMs have correctly predicted pairs or active compounds in benchmark calculations and prospective applications involving experimental evaluation [15].

## Scientific reasoning

Sequence-to-compound learning is an instructive example for a prediction task at the crossroads between computational feasibility and scientific reasoning. The idea to predict active compounds from protein sequences is not new, but it can now be addressed computationally in sophisticated ways using LMs. This section delves deeper into the underlying scientific challenges of LMs or other DL models designed for this purpose. In structure-based drug design, the availability of 3D target structure information enables the delineation of ligand binding sites and the application of computational approaches to identify candidate compounds with a high degree of shape and chemical complementarity to given sites. In three-dimensions, in the context of a folded protein structure, only a limited number of amino acid residues participate in ligand binding. These contact residues are typically widely distributed across the primary structure of the protein and might form characteristic sequence motifs for individual protein families. By contrast, the majority of residues in protein sequences are not involved in ligand binding but important for the structural integrity of a given protein fold. These residues might also form sequence motifs that are characteristic of secondary structure elements or other structural features. However, during evolution, protein structure has been much more conserved than sequence and large statistical variations are often observed in sequences adopting a given fold, up to the level that global sequence similarity is no longer detectable statistically.

Insights into the formation of ligand binding sites by a limited number of residues and statistical variations of sequences of proteins with similar structures are not available to a computational model learning sequence-to-compound mappings. Instead, the model learns to associate protein sequences with structures of active compounds based on large volumes of sequence and compound training data. Protein representation learning via PLMs might recognize characteristic patterns in sequences that are indicative of structural or functional features and produce informative embeddings. Consistent with our insights into protein sequence-structure relationships and ligand binding sites, one might hypothesize that a LM for sequence-to-compound predictions must be capable of learning residue patterns implicated in ligand binding to correctly predict novel compounds; an ambitious conjecture. Protein sequences can be modified through ‘computational mutations' and re-tested to identify residues that are important for correctly predicting an active compound [15]. In selected cases, such calculations might provide evidence for the importance of individual binding site residues for accurate predictions [15]. However, must this be the case? Can LMs only predict active compounds based on sequence data if binding site motifs are recognized? Or can predictions be driven by associating compound structures with residue patterns in global sequences that become statistical signatures although they are not implicated in ligand binding (potentially moving into Clever Hans territory…)? These questions point to critical issues. In sequence-to-compound modeling, LM predictions might not only produce promising results if a model indeed learns what we know to be determinants of specific protein-ligand interactions. There might be many other reasons for the success (or failure) of a model, which might be hidden to us. Ultimately, if predictions of events that have solid physical foundations are solely driven by statistical associations, LM or other DL models will strongly depend on training data and protocols and have limited generalization potential. Their predictions will not be sustainable. In this context, sound scientific reasoning requires awareness that ML might often not conform to our knowledge or pre-conceived notions and that stringent control calculations are required for hypothesis testing aiming to rationalize predictions. For sequence-to-compound modeling, systematic XAI analysis using alternative approaches will be essential for exploring the origins of prediction outcomes, avoid misinterpretation and critically judge model performance and most influential factors.

## Conclusion

Interpretation of ML models is of critical importance for interdisciplinary applications. However, the identification of features determining predictions must be distinguished from chemical or biological interpretability, for which feature attribution analysis provides a starting point. Furthermore, in life science and drug discovery applications, establishing causality for predictions often requires experimental follow-up. In general, special care must be taken to avoid ascribing prediction outcomes to incorrect reasons. Clearly formulated hypotheses that can be directly tested using a ML model often help to avoid such pitfalls. In pharmaceutical research, LM models offer many opportunities for addressing previously difficult or unfeasible prediction scenarios as machine translation tasks. Sequence-based compound design is an instructive example of a task that has become feasible through the use of LMs, but might be controversially viewed. Here, scientific reasoning becomes critically important at different levels, for example, by considering that positive predictions might not be a consequence of learning physical foundations of binding events, designing scientifically meaningful controls for such predictions, and avoiding premature interpretation of prediction results. Without doubt, for new predictions tasks tackled using LMs, the development of explanatory methods for analyzing learning characteristics of these models and origins of their predictions will become increasingly important.

## Editorial Board

J Bajorath is a member of the Future Science OA Editorial Board. They were not involved in any editorial decisions related to the publication of this article, and all author details were blinded to the article's peer reviewers as per the journal's double-blind peer review policy.
